# Time Dependent Pathway Activation of Signalling Cascades in Rat Organs after Short-Term Hyperoxia

**DOI:** 10.3390/ijms19071960

**Published:** 2018-07-04

**Authors:** Jochen Hinkelbein, Stefan Braunecker, Matthias Danz, Lennert Böhm, Andreas Hohn

**Affiliations:** 1 Department of Anesthesiology and Intensive Care Medicine, University Hospital of Cologne, 50937 Cologne, Germany; andreas.hohn@uk-koeln.de; 2 Department of Anaesthesiology, St George’s University Hospital, NHS Foundation Trust, London SW17 0QT, UK; stefan.braunecker@nhs.net; 3 Department of Anesthesiology, St. Martinus Hospital, 57462 Olpe, Germany; matthias.danz@gmx.de; 4 Emergency Department, University of Duesseldorf, 40225 Düsseldorf, Germany; lennert.boehm@gmx.de

**Keywords:** hyperoxia, oxygen, protein expression, proteomics, signaling cascades

## Abstract

Administration of oxygen is one of the most common interventions in medicine. Previous research showed that differential regulated proteins could be linked to hyperoxia-associated signaling cascades in different tissues. However, it still remains unclear which signaling pathways are activated by hyperoxia. The present study analyses hyperoxia-induced protein alterations in lung, brain, and kidney tissue using a proteomic and bioinformatic approach. Pooled data of 36 Wistar rats exposed to hyperoxia were used. To identify possible hyperoxia biomarkers, and to evaluate the relationship between protein alterations in hyperoxia affected organs and blood, proteomics data from brain, lung, and kidney were analyzed. Functional network analyses (IPA^®^, PathwaysStudio^®^, and GENEmania^®^) in combination with hierarchical cluster analysis (Perseus^®^) was used to identify relevant pathways and key proteins. Data of 54 2D-gels with more than 2500 significantly regulated spots per gel were collected. Thirty-eight differentially expressed proteins were identified and consecutively analyzed by bioinformatic methods. Most differences between hyperoxia and normoxia (21 proteins up-regulated, 17 proteins down-regulated) were found immediately after hyperoxia (15 protein spots), followed by day 3 (13 spots), and day 7 (10 spots). A highly significant association with inflammation and the inflammatory response was found. Cell proliferation, oxidative stress, apoptosis and cell death as well as cellular functions were revealed to be affected. Three hours of hyperoxia resulted in significant alterations of protein expression in different organs (brain, lung, kidney) up to seven days after exposure. Further studies are required to interpret the relevance of protein alterations in signaling cascades during/after hyperoxia.

## 1. Introduction

The application of oxygen is one of the most common interventions in medicine, both in pre-hospital and in-hospital settings. While the cellular oxygen supply represents one mainstay of survival, hyperoxia has a deleterious potential [1,2,3]. Recently, the risks of hyperoxia in critically ill and emergency patients have been intensively discussed [1,2,3,4,5]. While effects of hyperoxia on various organs (e.g., lungs, brain, or retina) have been investigated sufficiently in recent years [6,7], molecular effects, pathway alterations and affected signaling cascades in tissue or organs due to hyperoxia are rarely studied.

Oxygen toxicity is a long known phenomenon after exposure to high fractions of oxygen both in physiology [8] and clinical medicine [9]. Antoine Lavoisier described hyperoxia as early as 1789 in an animal model, which, however, did not return to interest until the middle of the 20th century. Today, hyperoxia gained significant interest during the post-resuscitation period. Here, increased levels of oxygen partial pressure lead to worse outcomes [2,3,4,5]. Furthermore, arterial hyperoxia may be associated with an increased mortality in critically ill patients [1].

However, the physiologic link between hyperoxia and tissue toxicity is not fully elucidated for all organs, but the analysis of changes in the pattern of protein expression could help to clarify the fundamental pathophysiology and reveal the affected signal transduction cascades that lead to hyperoxia-related organ dysfunction. For some specific organs, the effects of hyperoxia on the tissue were analyzed in detail (e.g., hyperoxia-induced lung injury mediated by inflammasomes [10,11], cysteine-rich protein 61 signaling [12], and β-catenin signaling [13]). Additionally, the role of the Caspases [14], first apoptosis signal receptor/cluster of differentiation **95** (Fas/CD-95) dependent signaling [15] and Caspase-1 dependent IL-1 and IL-18 [16] in hyperoxia induced brain injury are known.

Recent technological advances have made it possible to identify and quantify thousands of proteins in a single proteomics experiment. As a result of these developments, the analysis of data has become the bottleneck of proteomics experiments [17]. One major approach in understanding the negative consequences of hyperoxia is to analyze the patterns of protein expression in different tissues by using proteomics [18] and bioinformatic analyses [19,20,21]. To survey multiple differentially regulated proteins at the same time, a combination of two-dimensional gel electrophoresis (2D-PAGE), peptide mass fingerprinting via matrix-assisted laser desorption ionization time-of-flight mass spectrometry (MALDI-TOF-MS) and a bioinformatic molecular network analysis is used.

The hypothesis of this study was that there may be different alterations in protein expression after temporary hyperoxia identified when analyzing different organs. Additionally, the expression profile was analyzed to identify congruent protein changes in different organs. The aim of present study is to analyze changes in protein expression following a short period of hyperoxia in lung, brain, and kidney tissue of rats and to identify affected signal transduction cascades at different points in time.

## 2. Results

### 2.1. Expression of Proteins

Physiological parameters of the two groups differed only statistically significantly in the arterial oxygen partial pressure (Table 1). In the 54 2D-gels with an average of 2500 spots per gel, a total of 38 protein spots (brain: *n* = 9, lung: *n* = 12, kidney: *n* = 17) with a divergent protein expression were identified (Table 2) and were identified as 28 different proteins (brain: *n* = 9, lung: *n* = 10, kidney: *n* = 9).

### 2.2. Alterations in Protein Expression during Time

For the spots, most differences between the hyperoxia and normoxia groups were found immediately after hyperoxia (T0) (*n* = 15 significantly regulated protein spots), followed by T3 (*n* = 13 protein spots) and T7 (*n* = 10 protein spots). Twenty-one of the identified proteins showed up-regulation of synthesis, whereas 17 were down-regulated (Table 3, Table 4 and Table 5).

### 2.3. Ingenuity Pathway Analysis (IPA)

**Figure 1:**
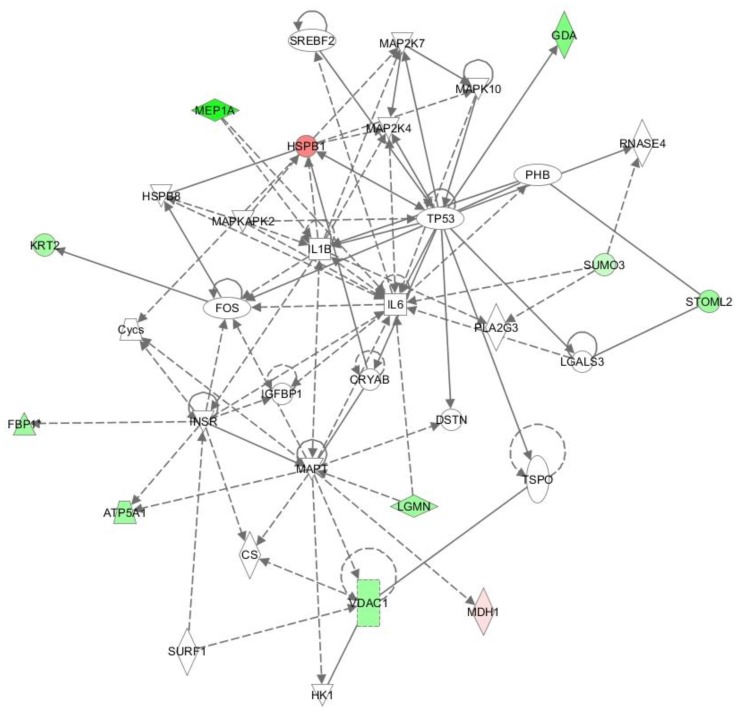
Ingenuity pathways analysis (IPA) functional network generated for data of NH0-, NH3-, and NH7-groups. Solid lines indicate direct interaction, dotted lines indicate indirect interaction. The identified proteins have in part direct (MEP1A), in part an adjacent influence on integrins (green/light green: up-regulation, red/light red: down-regulation).

Using IPA, all identified proteins (brain, lung and kidney) were examined for a possible molecular or functional relationship or interaction and the results are shown in a chart (Figure 1). The linked proteins showed the already known close relation of interleukin (IL-1, IL-6) and MAP-kinases.

**Figure 2:**
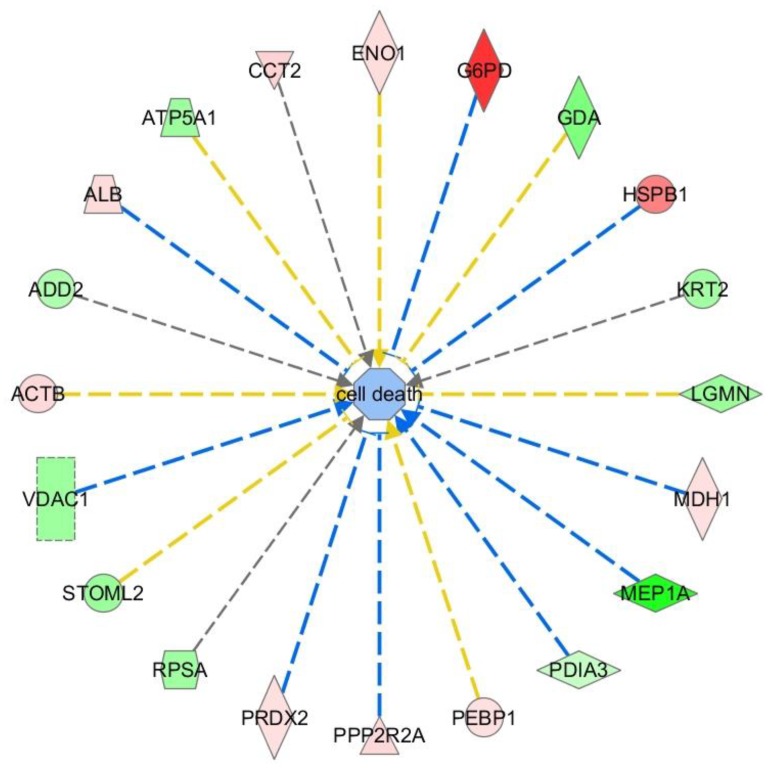
Proteins with influence on cell-death related signal transduction (green/light green: up-regulation, red/light red: down-regulation).

On the basis of known protein interactions, IPA showed highly significant correlation of the analyzed proteins with signaling cascades:

- Inflammation (inflammatory disease; *p* = 0.000000149; 11 proteins) and inflammatory response (inflammatory response; *p* = 0.000000149; 9 proteins).
- Apoptosis (cell death and survival; *p* = 0.000000189; 20 proteins) and cell death (*p* = 0.00000424; 20 proteins) (Figure 2).

### 2.4. Pathway Studio

**Figure 3:**
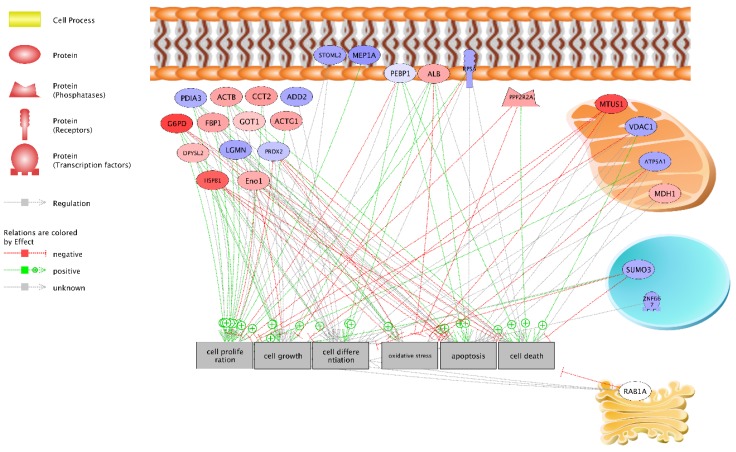
Interaction of proteins 3 days after hyperoxia. Pathway Studio (Elsevier, UK) (green arrow: positive regulation, red arrow: negative regulation).

Using pathway studio, we developed an overview of the cellular localization and function of the identified proteins (Figure 3). Thirteen proteins (PDIA3, ACTB, CCT2, ADD2, G6PD, FBP1, GOT1, ACTG1, DPYSL2, LGMN, PRDX2, HSPB1, ENO1) were localized in the cytosol, five on the plasmatic membrane (STOML2, MEP1A, PEBP1, ALB, RPSA), four in the mitochondria (MTUS1, VDAC1, ATPSA1, MDH1), one in the Golgi apparatus (RAB1A) and two in the vacuoles (SUMO3, ZNF667). One is a cytoplasmatic phosphatase (PPP2R2A). Twelve proteins were up-regulated, and 13 down-regulated.

Six different cellular functions affected by 3 h of hyperoxia were found: proliferation (stimulated), migration (stimulated), differentiation (stimulated), oxidative stress response (inhibited), apoptosis (even), cell-death (even).

### 2.5. GENEmania

**Figure 4:**
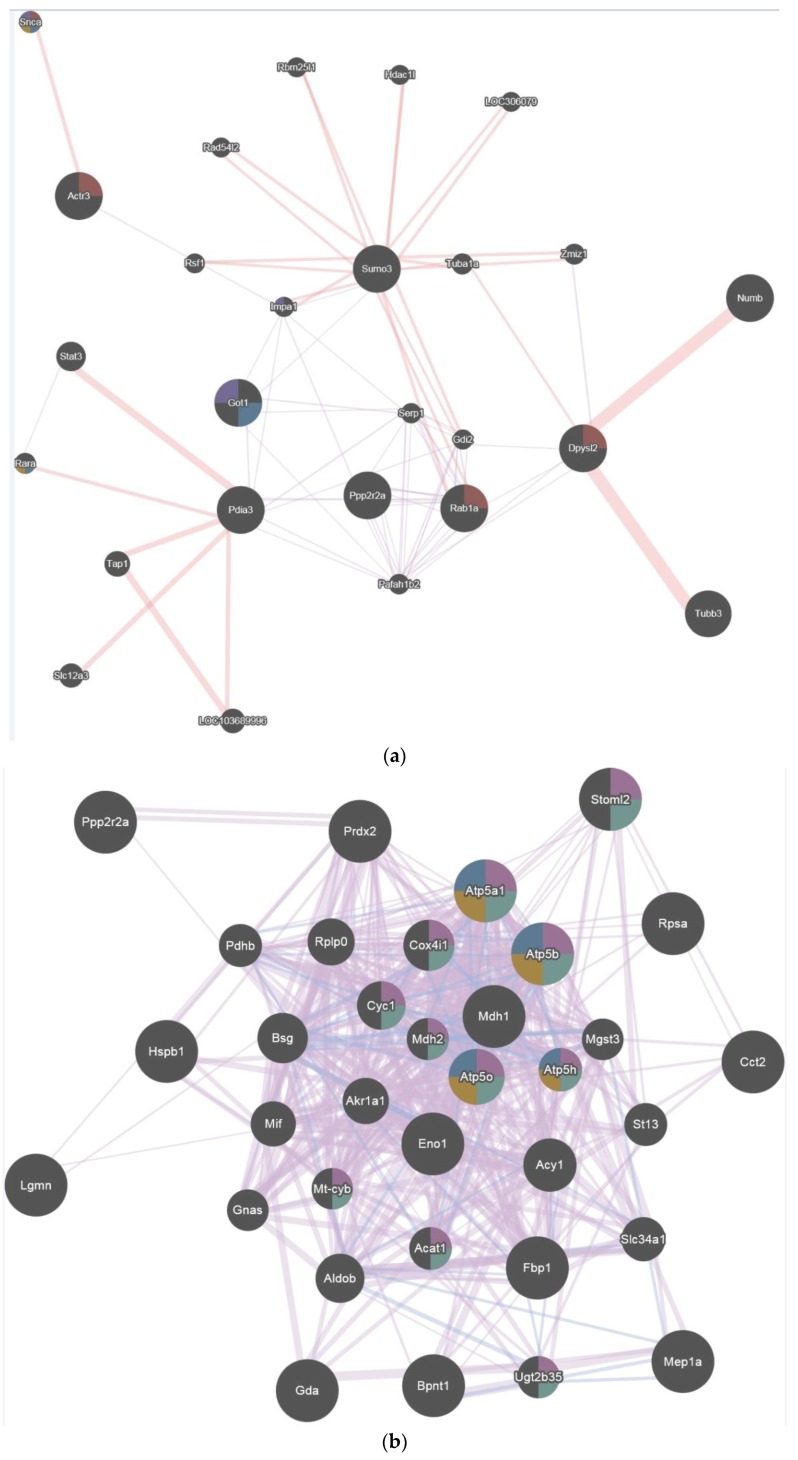

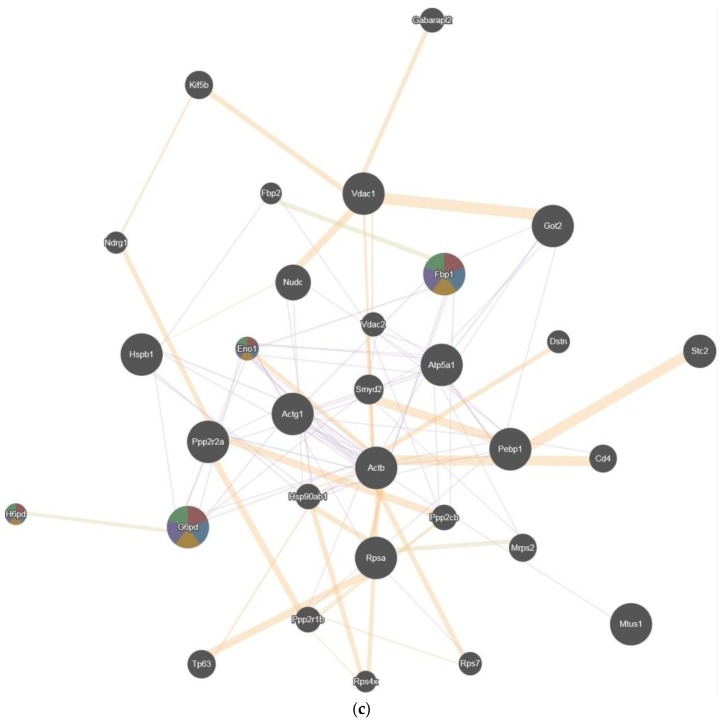
(**a**) GENEmania network with significantly regulated proteins involved at T0; (**b**) GENEmania network with significantly regulated proteins involved at T3; (**c**) GENEmania network with significantly regulated proteins involved at T7.

GENEmania revealed several associations between proteins and signaling pathways. Immediately after exposure (T0), significantly regulated proteins (Table 3) were found to be involved in carboxylic acid binding, polyol metabolic process, and cellular carbohydrate metabolic process (Figure 4a).

Three days after exposure (T3), significantly regulated proteins (Table 4) were found to be involved in mitochondrial processes, mitochondria proton transporting ATP synthase complex, and generation of precursor metabolites and energy (Figure 4b).

Seven days after exposure (T7), significantly regulated proteins (Table 5) were found to be involved in glucose catabolic process, glucose metabolic process, and carbohydrate catabolic process (Figure 4c).

### 2.6. Hierarchical Cluster Analysis

**Figure 5:**
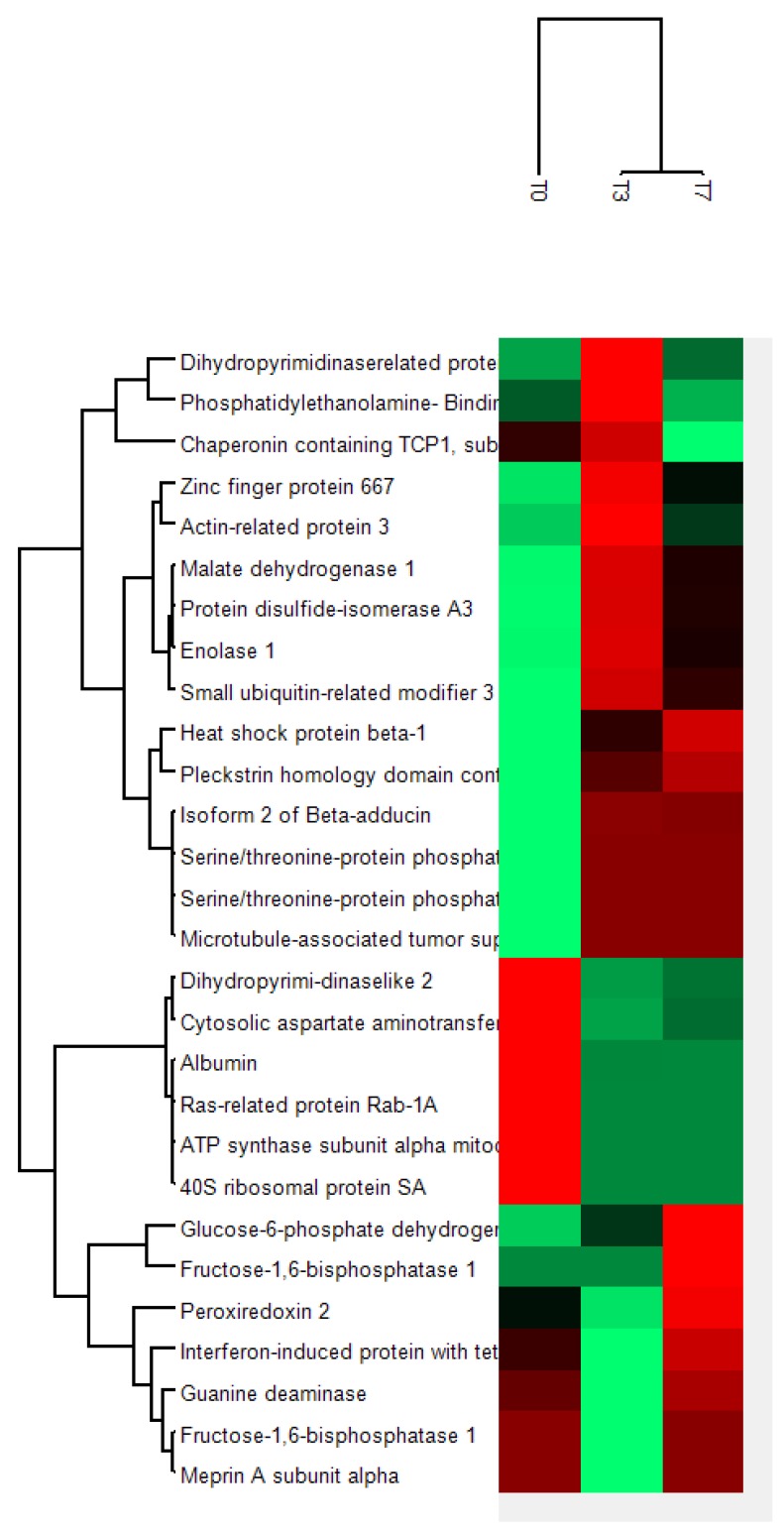
Heatmap generated by PERSEUS^®^. The groups T3 and T7 show comparable regulation and are, therefore, grouped together. Green fields indicate up-regulation and red fields down-regulation.

The groups T3 and T7 show comparable regulation (Figure 5) and are, therefore, grouped together. Subgroup analysis of clusters showed inflammation and inflammatory response as well as apoptosis (cell death and survival) cell growth to be related to the clusters.

## 3. Discussion

### 3.1. General Hyperoxia Considerations and Oxygen Toxicity

Oxygen toxicity follows exposure to increased oxygen partial pressure by the formation of highly reactive oxygen compounds (superoxides, hydrogen peroxide, hydroxy-radicals) [22]. Oxidative stress terms a condition in which the production of oxidants exceeds the organism’s anti-oxidative capacity, a disequilibrium inevitably followed by damage of the molecular cell compounds.

In general, hyperoxia leads to an overwhelming production of reactive oxygen species (ROS; •O_2_^−^) which in turn initiate an inflammatory cellular response. The degree of damage caused by hyperoxia depends on the balance between ROS production and the capacity of antioxidant systems in different organs [23,24]. A linear increase of ROS concentration with oxygen tension in mitochondria has been observed [25]. •O_2_^−^ is generated by the mitochondrial electron transport chain by the transfer of an electron to molecular O_2_.

Additionally, oxygen tends to build superoxides with particular metals, destroying double bonds in fatty acids. Even though the physical impact of oxidative reactions on proteins are well known, the molecular changes are still unknown in detail. Former studies could deduce that both short- and long-term oxygen delivery can lead to transient or permanent effects in different tissues [2,16,26,27]. But contemporary works brought equivocal results [28,29,30].

As another source of ROS, the nicotinamide adenine dinucleotide phosphate (NADPH) oxidase (NOX) family, utilizes NADPH/NADH as an electron donor to catalyze reduction of molecular oxygen to •O_2_^−^ at the extracellular side of the plasma membrane [31,32]. These hyperoxia-induced effects are mediated by complex sets of genes and their products rather than being the result of only one single gene.

The type and degree of ROS damage to organs and tissue has not been investigated in detail yet. There are some studies suggesting that high oxygen concentrations in inspired air as well as resulting hyperoxia may lead to detrimental organ effects [6,7,24,33,34]. However, results of previous studies on beneficial as well as detrimental effects of hyperoxia are still controversially discussed [28,35,36].

### 3.2. Protein Alterations in the Present Study

Changes in protein expression following 3 h of hyperoxia were detectable up to 7 days after exposure in the present study. Using methods and tools of proteomics and bioinformatic analysis and interpretations, the corresponding proteins were connected to oxygen toxicity.

## 4. Materials and Methods

### 4.1. Experimental Hyperoxia Model and Proteomic Data from Previous Studies

In three previous studies, a total of 36 rats weighing 280 ± 21 g were used and prepared for proteomic analysis [6,7,37]. These studies were approved by the review board for animal studies (Regierungspräsidium Karlsruhe, Germany; no. AZ 35-9185.81/G-56/04). For the present study, proteins found to be significantly altered in the previous studies were used and combined. Briefly, the following methodology was used: after weighing, 18 animals were randomly assigned to three normobaric hyperoxia groups (NH) and the remaining 18 rats were allocated to three normobaric normoxia groups (NN). Following this group formation, all animals (NH and NN) were randomly assigned to one specific subgroup: “immediate analysis” (NH0 or NN0), “3 days analysis” (NH3 or NN3, T3), or “7 days analysis” (NH7 or NN7, T7). This resulted in six different groups with six animals each.

At the beginning of the experiments, rats were placed into an air-sealed box (30 cm × 18 cm × 18 cm) with one small inlet hole and one small outlet hole. For animals receiving hyperoxia, an oxygen line was connected to the inlet providing a flow of 5 L/min of pure medical oxygen (C99.5%; medical^®^, Air Liquide, Düsseldorf, Germany). This model resulted in an oxygen concentration of >95% within 3–5 min. Rats of the normoxia groups received 5 L/min room air until the end of the experiments. Oxygen concentration was measured continuously in both groups. After 3 h of hyperoxia or normoxia, experiments were terminated and the animals were quickly removed from the box. The rats of the NH3, NH7, NN3, and NN7 group were placed back into their cages and provided with food and water ad libitum until the planned end of the experiments (3 or 7 days after the experiments).

At the defined end-points of the experiments, the animals of the specific group (immediately, 3 or 7 days after exposure to 3 h of hyperoxia) were quickly anesthetized with sevoflurane in a concentration of 5% in room air and underwent both cardiac puncture for blood removal for analysis of arterial blood gases, electrolytes, hemoglobin (Bayer Vital Diagnostics, Rapidlab 865; Fernwald, Germany), blood cell count (Advia 60; Bayer Vital Diagnostics; Fernwald, Germany), albumin, and phosphate (Advia 2400; Bayer Vital Diagnostics; Fernwald, Germany). After blood removal, euthanasia was performed by decapitation. The brain, lung and kidneys were then removed for proteomic analysis as quickly as possible, frozen in isopentane prechilled to −40 to −50 °C, and stored at −80 °C until further analysis. Time from the end of the experiments to cardiac puncture as well as euthanasia was kept as short as possible to prevent rapid alterations in organ proteins and resumption to normoxia.

### 4.2. Proteomic Analysis

Two-dimensional gel electrophoresis was performed on rat brain essentially as described before [16,17,18,20,26]. All gels were run in triplicate to minimize inter-gel variability [38]. Gels destined for image analysis were silver stained after protein separation [31], digitalized, and the images analyzed using the PhoretixTM 2D Expression platform (NonlinearDynamics; Newcastle-upon-Tyne, UK). Spots representing proteins or their isoforms were defined as being circumscribed protein accumulations exhibiting a signal-to-background ratio surpassing at least 1.15, and their (spot) volumes were calculated from the grey values. Each individual spot was then matched to a corresponding spot in a reference gel, which was created as a virtual PC-generated averaged gel, and normalized spot volumes were yielded by relating the spot area to the total spot volume of all detected spots. Finally, induction factors (IF, expression factors) were compounded for each spot (protein) by dividing the mean normalized spot volume in the experimental group by the mean normalized spot volume in the control group. An IF = 2.5, for example, indicates a 2.5-fold increase in the expression of a protein in one group versus the other, a value of 0.5 indicates a 2.0-fold decrease. For statistical analysis, the normalized spot volumes were compared between spots in both groups NH and NN at each of the three time points using analysis of variance (ANOVA) with the Bonferroni correction, where statistical significance was set to *p* < 0.01. The *p*-value of <0.01 was set intentionally to identify relevant changes and to have an extremely high chance that protein alterations are not random.

Mathematical differences in the expression of a protein were considered biologically significant only if the arbitrary condition 0.5 > IF > 2.0 was fulfilled. Thereby, a protein abundance that was either doubled or halved made its changes more likely to affect cellular function. All differentially regulated spots subjected to a protein identification by peptide mass fingerprinting in the next step had to meet at least one condition: *p* < 0.01 or 0.5 > IF > 2.0.

**Figure 6:**
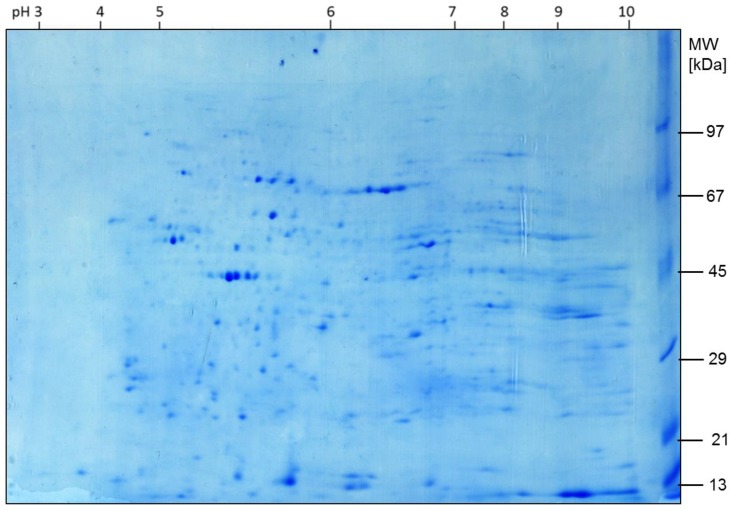
Sample gel of the control group at 3 days after hyperoxia (staining: Coomassie blue).

For spot identification, gels with a protein load of 250 µg were stained with colloidal Coomassie blue and matched to a silver stained reference gel (Figure 6). Differentially regulated spots which were detected in each triplicate were then excised from the Coomassie gel, in-gel digested with trypsin, and subjected to peptide mass fingerprinting via matrix-assisted-laser-desorption/ionization time-of-flight mass spectrometry (MALDI-TOF-MS). Database mining candidates with a Mascot score of more than 63 were considered statistically significant identifications at *p* < 0.05. In contrast to the mathematical spot volume calculations (*p* < 0.01), the *p*-value for database mining was set to <0.05 since this is the standard for bioinformatic identification of proteins by using MALDI-TOF. Significantly altered proteins were identified by mass spectrometry (MALDI-TOF MS) and used for further bioinformatic analysis to identify underlying networks, signaling cascades, and pathways affected.

### 4.3. Stepwise Bioinformatic Approach

In summary, as a first step, statistically significant regulated proteins were identified and analyzed by network analyses (Ingenuity Pathways Analysis (IPA®; QIAGEN; Redwood City, CA, USA), PathwayStudio^®^ (Elsevier, Amsterdam, The Netherlands), and GeneMANIA^®^ (Toronto, ON, Canada)). Afterwards, relevant proteins were grouped (groups: immediate, after 3 days, and after 7 days) using a hierarchical cluster analysis (Perseus^®^; MPI of Biochemistry, Martinsried, Germany). As a third step, proteins of similarly early up-regulated clusters underwent further network analysis to evaluate possible corresponding proteins or functions in blood and organ tissues. This approach to dealing with pooled proteomic data is described in detail below.

### 4.4. Ingenuity Pathway Analysis

The biochemical and functional interrelations of all significantly regulated proteins were analyzed using the “Ingenuity Pathway Analysis” (IPA)-Platform. (version 3.1, Ingenuity Systems Inc., Redwood City, CA, USA), a web-based application using published data from over 24,000 genes and corresponding proteins. Firstly, the differentially regulated proteins (focus proteins) were uploaded and used as starting points for building biological networks. Based on relevant protein–protein interactions known from the published data, IPA then determined the interactions between the focus proteins and all other proteins in the IPA-associated database.

After mapping, IPA calculated a score for the network according to the fit of the protein set which quantifies the probability of the focus proteins being found together in a network at random; values above 2.0 reflect a confidence of at least 99% for the network map not having been produced coincidentally. Finally, biological functions assigned to each network were ranked according to the significance of that function to the network.

### 4.5. Pathway Studio

Pathway Studio (Elsevier, Elsevier, Amsterdam, The Netherlands) is a supportive tool that enables the analysis and visualization of gene expression, disease mechanisms, proteomics and metabolics. It lends the possibility to visualize the effect of changing protein expressions on the pathomechanism of selected diseases at a cellular level, again using already published data.

### 4.6. Network Analysis of Proteins (GeneMANIA^®^)

Biological functions of relevantly regulated proteins were identified using functional network analysis. GeneMANIA^®^ (available online: http://www.genemania.org/) is a tool that helps to predict interactions and function of genes in terms of network and, when available, of pathway [20,22]. It gives the possibility of customizing the network, and allows choosing data sources or highlight specific functions, with a more comfortable graphic experience [20]. It is developed and continually updated by the University of Toronto and is funded by the Ontario Ministry of Research and Innovation.

GeneMANIA^®^ knowledge is based on data from large databases, which includes Gene Expression Omnibus, BioGRID, EMBL-EBI, Pfam, Ensembl, Mouse Genome Informatics, the National Center for Biotechnology Information, InParanoid, and Pathway Commons [20,22]. It was developed for making predictions about gene or protein function based on a query of list of proteins that share a function of interest. The software allows researchers to take advantage of the persistent improvement and proliferation of high-throughput genomics and proteomics data sets by making up-to-date predictions of their interaction with other genes or proteins [20,22].

As these software programs use different algorithms, we decided to perform the bioinformatic analyses with all of them in order to retrieve the highest number of predicted interactions, maintaining an acceptable level of confidence. The associated functions detected by the software were downloaded in TAB-separated-values format and exported to Microsoft Excel^®^ (Microsoft, Redmond, WA, USA; version 2007) where they were filtered in subgroups which were reanalyzed using GeneMANIA^®^.

### 4.7. Hierarchical Cluster Analysis

Heat maps are an efficient method of visualizing complex data sets organized as matrices [39]. Perseus^®^ (Max-Planck-Institute of Biochemistry, Martinsried, Germany; version 1.5.8.5) is a holistic software platform that allows continuous expansion of scalable analytical tools, their smooth integration and reusability while providing the user with explicit documentation of the analysis steps and parameters [40]. Quantitative information concerning proteins that altered relevantly their expression at 0, 3, and 7 days after hyperoxia was converted to TSV (Tab-Separated Values) text file using Microsoft Excel^®^ (Microsoft, Redmond, WA, USA; version 2007). Each value was reported as fold change in comparison to the sham group values, so that a positive number represents a higher expression of a spot at 0, 3, and 7 days while a negative number represents lower expression of a spot at 0, 3, and 7 days. In this format the data were analyzed using the free software Perseus^®^ (Max-Planck-Institute of Biochemistry, Martinsried, Germany; version 1.5.8.5) which performed the *Z*-scoring and, consequently, the hierarchical cluster analysis. The resulting heat map can be interpreted on the basis of color intensity. In our case, a red brick represents a protein whose expression at a particular time was increased when compared to the value of the same protein in the sham group at that time.

### 4.8. Identification of Regulation Pathways and Biomarker Candidates

On the basis of the cluster analysis, further subgroup network analyses of similarly up-regulated proteins in the groups analyzed were performed to find regulation patterns and identify possible biomarkers.

### 4.9. Statistical Analysis

For the statistical analysis of blood gas data, the student´s *t*-test was used. A *p*-value of 5% (*p* < 0.05) was considered statistically significant. For statistical analysis of spot volumes, the normalized spot volumes were compared between spots in both groups using analysis of variance (ANOVA) with Bonferroni correction, where statistical significance was set to *p* < 0.01. The *p*-value of <0.01 was set intentionally to identify relevant changes and to have an extremely high chance that protein alterations are not by random. In contrast to the mathematical spot volume calculations (*p* < 0.01), the *p*-value for database mining was set to <0.05.

## 5. Specific Proteins Involved

### 5.1. Meprin A (MEP1A)

Meprin A (MEP1A) is a metalloendopeptidase and belongs to the family of astacins. Investigations showed that MEPA1 is capable of breaking down extracellular matrix proteins, processing inflammatory cytokines, and is deemed to aid in the understanding of a variety of diseases, from inflammatory response to acute renal failure [41]. Besides the fact that Meprin A could be useful as a biomarker for renal failure, little information is known about the role of MEP1A in the pathogenesis of severe kidney disease. In our study, MEP1A showed a significant down-regulation at T3 (IF = −4.17). Although little insight exists into a decreased expression of Meprin A, a redistribution of the protein is associated with the presence of a cell damage and inflammatory response [42]. The reduced expression of Meprin A in the present study is a clear indication of a possible link between hyperoxia and a cellular response of the kidney.

### 5.2. Voltage-Dependent Anionselective Channel Protein 1 (VDAC1)

Voltage-Dependent Anionselective Channel Protein 1 (VDAC1) is a protein of the outer mitochondrial membrane and is crucial for the regulation of the mitochondrial metabolism. It acts as a point of convergence for various signals for cell death or cell survival [38]. It is also a major player in apoptosis and is involved in the release of cytochrome C and interactions with other anti-apoptotic proteins. The reduction in VDAC1 expression on T3 and T7 (IF = −1.81) could indicate an involvement of the mitochondrial metabolism in the cellular changes following hyperoxia. In addition, in the IP analysis VDAC1 was associated with cell death, cell proliferation and necrosis.

### 5.3. Peroxiredoxin 2 (PRDX2)

Peroxiredoxin 2 (PRDX2; natural killer cell enhancing factor B), which was significantly down-regulated in the present study 3 days after exposure to normobaric hyperoxia (e.g., IF = −4.00 after 3 days; Table 2), is one member of the peroxiredoxin family and is predominantly localized in the cytosol [26]. It is a dimeric protein with an amino acid sequence highly conserved in all eukaryotic species [29] and is exclusively expressed in neurons, but not in other cells of the brain [26]. In addition, PRDX2 is expressed, e.g., in blood cells. Proteins of the peroxiredoxin gene and other antioxidant gene families have been documented to exert antioxidant and cytoprotective effects [43]. Hence, the decreased expression of PRDX2 in the present study could represent a cellular response facilitating apoptosis which is in accordance with data from Taglialatela et al. [27], suggesting that hyperoxia leads to an increased apoptotic turnover in the rat brain.

### 5.4. Glucose-6-Phosphate Dehydrogenase (G6PD)

In the present study, glucose-6-phosphate dehydrogenase (G6PD) expression was massively up-regulated in the rat brain 7 days following oxygen exposure (IF = 23.91). The enzyme is located in the cytoplasm and catalyzes the first rate-limiting step in the pentose phosphate pathway. In addition, it can mediate generation of peroxynitrite (from nitric oxide and the superoxide radical) and protect against nitric oxide-mediated apoptosis in neurons [30]. In the present study, the up-regulation of this enzyme may indicate a reactive counter mechanism against hyperoxia-generated superoxides that inhibit glycolysis and thus energy production in the brain [30]. Regarding up-regulation of G6PD, it is of significance that this is upregulated in the brain at all time intervals, which may reflect changes in glucose due to anesthesia as G6PD is rapidly induced by hypoxia, suggesting or confirming that the animals were hypoxic due to anesthesia immediately before euthanasia.

### 5.5. Enolase 1 (ENO1)

Enolase 1 (ENO1) was up-regulated 3 days after hyperoxia (IF = 1.54). It is a multifunctional glycolytic enzyme that plays various roles including in inflammation, growth control, and allergic responses [44]. With its role in hypoxia tolerance, ENO1 is expressed in the cytoplasm of neurons and can clinically be used to estimate brain injury in intensive care medicine—its up-regulation may thus reflect the occurrence of some form of neuronal injury after normobaric hyperoxia.

### 5.6. Dihydropyrimidinase 2 (DYPSL2)

Dihydropyrimidinase 2 (DYPSL2, CRMP2) is a membrane-associated protein which belongs to the collapsin response mediator proteins (CRMPs) that are believed to play a crucial role in neuronal differentiation, axonal guidance, and neuronal outgrowth [45]. DYPSL2 is implicated in the regulation of intracellular signaling pathways [46] and was up-regulated in the present study (IF = 4.09 immediate after hyperoxia). This protein is abundant in the nervous system, especially during development and it remains expressed in the adult brain, suggesting that the process of axonal outgrowth is important as mechanism of repair and regeneration of adult neurons [47]. Up-regulation of DYPSL2 in the present study during hyperoxia suggests a possible role for neuronal injury repair.

### 5.7. Heat Shock Protein beta-1 (HSPB1)

Heat shock protein beta-1 (HSPB1) belongs to the family of ubiquitous and abundant expressed stress proteins (small heat shock proteins, sHSPs). During oxidative stress, HSPB1 increases cell survival in response to apoptotic stimuli by inhibiting caspase activation [47], functions as a molecular chaperone refolding non-native proteins, prevents stress-induced disruption of the cytoskeleton by stabilizing F-actin filaments [48], and protects against ROS generated through oxidative stress. During NH as a sufficient stress factor, we could detect an up-regulation of HSPB1 over the whole period of investigation (IF = 6.79; Table 2).

### 5.8. Protein Disulfide-Isomerase A3 (PDIA3)

Protein disulfide-isomerase A3 (PDIA3) is mainly present in the endothelial reticulum (ER) but can also be found in the nucleus, extracellular space, cytosol, and on the cell surface. PDIA3 forms complexes with calreticulin and calnexin in the ER and functions as part of the glycoprotein-specific quality assurance in the lumen of the ER [49]. In our study, PDIA3 is down-regulated after NH (IF = −3.06). This result is concordant to previous results from Xu et al. [50]. They demonstrated that DIA3 was significantly decreased in neonatal rat lung tissue after a prolonged hyperoxic exposure, suggesting PDIA3 might be associated with hyperoxia-induced neonatal lung injury as it helps to modulate ER stress-induced apoptosis [50].

### 5.9. b-Adducin 2 (ADD2)

Beta-adducin (ADD2, ADDB) is widely expressed in multiple tissues like erythrocytes, brain, and lung. Its role as a structural protein in erythrocytes is well understood: a lack of ADD2 leads to decreased deformability and increased osmotic fragility [51]. A comparable structural function in lung tissue is not yet demonstrated, but the dramatic down-regulation of ADD2 as a result of short time hyperoxia (IF = −92.94; Table 2) may indicate a role in pulmonary stress response. Since ADD2 is involved in keeping up cell configuration, a participation in endothelial structural integrity is thoroughly possible.

### 5.10. Organ Effects

In the lung, hyperoxia causes a massive production of reactive oxygen species (ROS), which in turn initiate inflammatory response, destruction of the alveolar-capillary barrier, and impaired gas exchange [52,53]. The excessive production of ROS under hyperoxic conditions leads to modifications of macromolecules and pulmonary cell death [54,55].

Besides pulmonary complications, cerebral effects during hyperoxia remain to be a focus of prominent interest today, as oxidative metabolism and the generation of ROS play a major role in the central nervous system (CNS) [56,57]. In the brain, hyperoxia causes a transient but significant decrease in cerebral blood flow (CBF) [58], followed by a later rise [59], and its major biological effects include an induction of molecular stress responses, inflammation, as well as modulation of cell death and cell growth [6,60]. Also in the brain, NFκB serves as a central mediator of stress and is inhibited by hyperoxia [6] which also was demonstrated to be associated with inflammation, Alzheimer´s disease, oxidative stress, apoptosis, and cell death as well as cell growth, survival and differentiation pathways [6].

### 5.11. Interpretation of Changes in Protein Expression

Most of the signal transduction cascades identified by bioinformatic analysis are associated with a stress response, cellular toxicity, and inflammatory response. The effects of a short-term hyperoxia were on signal transduction were consistent over all organs. By IPA, the identified proteins showed in part a direct (MEP1A), in part a more adjacent effect on interleukins (IL-6 and IL-1), with especially IL-6 being a central part of systemic inflammation [61,62]. Additionally, IL-1 and IL-6 take part in proliferation and differentiation of multiple cell types [63,64]. Furthermore, IL-6 could be linked to apoptosis [65], so as to influence cellular integrity.

Besides, we found different mitogen activated protein kinases (MAP-K) being associated with proteins induced by temporal hyperoxia. As part of signal transducing processes, the MAP-Ks belong to stress-reaction [29] and facilitate an apoptosis reaction via extracellular signal-regulated kinases (ERKs) [66,67], whereas the association between hyperoxia, MAP-Ks and apoptosis has been shown before [68].

### 5.12. Bioinformatic Analyses

Interestingly, most differences were found immediately after hyperoxia exposure (15 spots) decreasing over time until day 7. However, it is not clear from the present point of view if the hyperoxic protein regulation alterations are limited to the first few days after exposure or if the alterations last longer.

Interactions of superoxide dismutases (SODs) with reactive oxygen species (ROS) have important roles during hyperoxia [69]. ROS toxicity has also been shown to be a common feature underlying some respiratory diseases by initiating inflammatory response, destruction of the alveolar-capillary barrier, and impaired gas exchange [52,53].

Using the bioinformatic networks described, six different figures (Figure 2, Figure 3, Figure 4 and Figure 5c) were generated explaining the function of proteins identified within the network of other un-identified proteins. These figures give insight into details of the network and facilitate detailed understanding of the function of these proteins.

Besides the classical network, IPA identified 20 proteins to be associated with cell death (Figure 3) which is in agreement to results of the other analyses. Also pathway studio (Figure 4) found an association to cell death, oxidative stress, apoptosis, cell proliferation/differentiation, and cell growth.

Using GENEmania (Figure 5a–c), proteins identified were part of a network related to other proteins after hyperoxia exposure. Immediately after hyperoxia, proteins were involved in organelle localization, carboxylic acid binding, monocarboxylic acid binding, and polyol metabolic process. Three days after exposure they were linked to organelle inner membrane, mitochondrial inner membrane, mitochondrial proton-transporting ATP synthase complex, and proton-transporting ATP synthase complex as well as to the glucose catabolic process, monosaccharide catabolic process, hexose catabolic process, carbohydrate catabolic process, and single-organism carbohydrate catabolic process 7 days after exposure.

### 5.13. Limitations

Using the molecular techniques presented in the present study has certain limitations.

The time from the end of the experiments to cardiac puncture as well as euthanasia was kept as short as possible to prevent rapid alterations in organ proteins and resumption to normoxia. However, there is a chance of rapid alterations of protein expression during that time. Furthermore, exposure to anesthetic agents and the time it takes to achieve appropriate anesthetic depth for cardiac puncture will have a significant impact.

Since only low abundant protein spots were found to have changed after exposure to hyperoxia, interpretation of data has to be done carefully. It is always possible that signals may originate from artificial background staining and this can be easily missed if the signal is weak. Since the protein samples are very complex and the high and medium abundant proteins did not seem to be affected by the treatment, a reduction of the complexity could be considered.

Separation of proteins in 2D gels reflecting only a smaller window of the proteome, by e.g., using narrower pH gradients, could reduce sample complexity. However, using a smaller window may veil other proteins at the border of possible detection (e.g., high or low pH or high or low molecular mass).

Furthermore, proteins in the sample were analyzed by MALDI-TOF only. Of course, this method uses statistics to identify the protein by analyzing protein fragments (i.e., peptides). To identify the specific proteins, the MASCOT score is used which is based on statistical methods. Thus, it cannot be ruled out that a protein could be misidentified. Therefore, some authors use additional methods (e.g., Western blot, ELISA) to proof their results. This was not possible in the present study since we did not have any protein material left. Finally, there might be (theoretically) the risk that not all proteins were identified by MALDI-TOF correctly.

Besides the mentioned limitations, there are major differences between human and rat hemoglobin. Rat Hb chains differ by at least 55 residues from those of human Hb chains, including differences in the heme pockets. To correctly determine rat blood gases, human blood gas analyzers should not be used since it is calibrated for human Hb. A more accurate method is the use of an Abaxis VetScan which is calibrated for rat Hb.

Last, anesthesia depresses breathing and blunts the ventilator responses to CO_2_ possibly causing hypercapnia in the animal model used. Anesthesia also reduces the sensitivity of carotid and aortic body chemoreceptors to hypoxia. Euthanasia of animals by excessive anesthetics can result in hypoxia and major changes in blood gases as well as genes involved in the hyperoxia responses. Therefore, some anesthesia-induced protein alterations cannot be ruled out.

## 6. Conclusions

The results of the present study satisfactorily show a relevant change in protein expression after 3 h normobaric hyperoxia that remains detectable for up to 7 days. Bioinformatic pathway analysis indicates a close relation of hyperoxia with cell-growth, regulation of apoptosis and inflammatory reactions.

The study’s experimental protocol did not intend to identify diagnostic markers to quantify hyperoxia-induced organ injury, but instead aimed to detect and understand both changes in protein expression and molecular pathways affected by normobaric hyperoxia in rat tissues, acknowledging the problem of only limited transferability of the results to the human. New clinical research can elucidate the aftermath of medically-induced normobaric hyperoxia in intensive and anesthesiology care.

## Figures and Tables

**Table 1:** Physiological parameters (hyperoxia group versus normoxia groups) immediately after normobaric hyperoxia exposure (3 h of 1.0 FiO_2_).

Parameter	Hyperoxia-Group NH_0_ (*n* = 6)	Normoxia-Group NN_0_ (*n* = 6)	Significance Level
**Blood Gases**			
Oxygen partial pressure (pO_2_, mmHg)	580 ± 49	89 ± 9	*p* < 0.05
Carbon dioxide partial pressure (pCO_2_, mmHg)	38.1 ± 5.2	36.4 ± 3.9	n.s.
HCO_3_- actual (HCO3^−^-act, mmol/L)	24.5 ± 5.0	26.0 ± 3.3	n.s.
Plasma albumin (g/L)	36.0 ± 1.3	36.3 ± 1.0	n.s.
**Electrolytes**			
Glucose (mg/dL)	191 ± 28	188 ± 30	n.s.
Lactate (mmol/L)	2.0 ± 1.7	2.2 ± 2.0	n.s.
**Blood Cells**			
Leukocytes (10^9^/L)	8.45 ± 2.78	8.83 ± 2.97	n.s.
Thrombocytes (10^9^/L)	709 ± 99	792 ± 111	n.s.
**Circulation**			
Heart rate (1/min)	402 ± 78	452 ± 44	n.s.
Body temperature (°C)	37.6 ± 0.5	37.9 ± 0.4	n.s.
**Other**			
Weight (g)	286 ± 23	274 ± 16	n.s.

**Table 2:** Significantly regulated proteins in different tissues (brain, lung, and kidney). Significant induction factors (IF) are printed red. Up-regulation is expressed by a positive number and down-regulation by a negative number.

Organ	Molecular Weight (kDa)	Significance, *t*-Test	Protein Mascot Score	Protein Name	Accession Number	IF Day 0	IF Day 3	IF Day 7
Kidney	72	*p* = 0.012	71	Meprin A subunit alpha	MEP1A_RAT		−4.17	
Kidney	70	*p* = 0.022	23	Microtubule-associated tumor suppressor 1 homolog	MTUS1_RAT		8.12	8.12
Kidney	33	*p* = 0.035	90	40S ribosomal protein SA	RSSA_RAT	−1.77	−1.77	−1.77
Kidney	29	*p* = 0.011	202	Fructose-1,6-bisphosphatase 1	F16P1_RAT		−1.87	
				Stomatin-like protein 2, mitochondrial	STML2_RAT			
				3′(2′),5′-bisphosphate nucleotidase 1	BPNT1_RAT			
				Legumain	LGMN_RAT			
Kidney	28	*p* = 0.03	64	ATP synthase subunit alpha mitochondrial	ATPA_RAT		−1.81	−1.81
				Voltage-dependent anionselective channel protein 1	VDAC1_RAT			
Kidney	28	*p* = 0.042	256	Fructose-1,6-bisphosphatase 1	F16P1_RAT			1.74
				Actin, cytoplasmic 2	ACTG_RAT			
				Actin, cytoplasmic 1	ACTB_RAT			
Kidney	23	*p* = 0.018	60	Serine/threonine-protein phosphatase 2A 55 kDa regulatory subunit B alpha isoform	2ABA_RAT	1.74	1.74	1.74
Kidney	23	*p* = 0.012	60	Serine/threonine-protein phosphatase 2A 55 kDa regulatory subunit B alpha isoform	2ABA_RAT	1.74	1.74	1.74
Kidney	14	*p* = 0.033	59	Ras-related protein Rab-1A	RAB1A_RAT	1.81		
Brain		*p* < 0.001	310	Cytosolic aspartate aminotransferase	GOT1	8.95	1.15	2.13
Brain		*p* < 0.01	116	Peroxiredoxin 2	PRDX2	−1.35	−4.00	−1.35
Brain		*p* < 0.001	256	Glucose-6-phosphate dehydrogenase	G6PD	2.42	9.46	23.91
Brain		*p* < 0.01	115	Phosphatidylethanolamine- Binding protein 1	PEBP1	−2.78	−1.07	−4.54
Brain		*p* < 0.01	318	Enolase 1	ENO1	−1.54	1.54	1.17
Brain		*p* < 0.001	342	Albumin	ALB	13.25	1.59	1.53
Brain		*p* < 0.001	205	Dihydropyrimi-dinaselike 2	DPYSL2	4.09	1.32	1.59
Brain		*p* < 0.001	135	Chaperonin containing TCP1, subunit 2 (β)	CCT2	1.73	2.04	1.13
Brain		*p* < 0.01	131	Malate dehydrogenase 1	MDH1	−1.23	1.43	1.18
Lung		*p* < 0.001		Interferon-induced protein with tetratricopeptide repeats 3	IFIT3	1.37	−1.62	2.72
Lung		*p* < 0.001		Heat shock protein beta-1	HSPB1	3.87	5.77	6.79
Lung		*p* < 0.01		Actin-related protein 3	ACTR3	−4.93	−1.73	−3.92
Lung		*p* = 0.148		Guanine deaminase	GDA	−1.52	−2.40	−1.36
Lung		*p* < 0.01		Protein disulfide-isomerase A3	PDIA3	−3.06	−1.05	−1.84
Lung		*p* < 0.01		Dihydropyrimidinaserelated protein 2	DPYSL2	−2.27	−1.21	−2.13
Lung		*p* < 0.01		Zinc finger protein 667	ZNF667	−3.96	−1.51	−2.85
Lung		*p* < 0.01		Isoform 2 of Beta-adducin	ADD2	−92.94	−1.51	−3.10
Lung		*p* < 0.01		Small ubiquitin-related modifier 3	SUMO3	−8.53	−1.05	−3.66
Lung		*p* < 0.01		Pleckstrin homology domain containing, family A member 7	PLEKHA7	−8.29	−3.13	−1.77

**Table 3:** Significantly regulated proteins immediately after hyperoxia (T0) in different organs. Positive values express up-regulation (*n* = 7), negative values down-regulation (*n* = 8).

Organ	Protein Name	Accession Number	IF at T0
Kidney	40S ribosomal protein SA	RSSA_RAT	−1.77
Kidney	Serine/threonine-protein phosphatase 2A 55 kDa regulatory subunit B alpha isoform	2ABA_RAT	1.74
Kidney	Serine/threonine-protein phosphatase 2A 55 kDa regulatory subunit B alpha isoform	2ABA_RAT	1.74
Kidney	Ras-related protein Rab-1A	RAB1A_RAT	1.81
Brain	Cytosolic aspartate aminotransferase	GOT1	8.95
Brain	Albumin	ALB	13.25
Brain	Dihydropyrimi-dinaselike 2	DPYSL2	4.09
Lung	Heat shock protein beta-1	HSPB1	3.87
Lung	Actin-related protein 3	ACTR3	−4.93
Lung	Protein disulfide-isomerase A3	PDIA3	−3.06
Lung	Dihydropyrimidinaserelated protein 2	DPYSL2	−2.27
Lung	Zinc finger protein 667	ZNF667	−3.96
Lung	Isoform 2 of Beta-adducin	ADD2	−92.94
Lung	Small ubiquitin-related modifier 3	SUMO3	−8.53
Lung	Pleckstrin homology domain containing, family A member 7	PLEKHA7	−8.29

**Table 4:** Significantly regulated proteins 3 days after hyperoxia (T3) in different organs. Positive values express up-regulation (*n* = 7), negative values down-regulation (*n* = 6).

Organ	Protein Name	Accession Number	IF at T3
Kidney	Meprin A subunit alpha	MEP1A_RAT	−4.17
Kidney	Microtubule-associated tumor suppressor 1 homolog	MTUS1_RAT	8.12
Kidney	40S ribosomal protein SA	RSSA_RAT	−1.77
Kidney	Fructose-1,6-bisphosphatase 1	F16P1_RAT	−1.87
	Stomatin-like protein 2, mitochondrial	STML2_RAT	
	3′(2′),5′-bisphosphate nucleotidase 1	BPNT1_RAT	
	Legumain	LGMN_RAT	
Kidney	ATP synthase subunit alpha mitochondrial	ATPA_RAT	−1.81
	Voltage-dependent anionselective channel protein 1	VDAC1_RAT	
Kidney	Serine/threonine-protein phosphatase 2A 55 kDa regulatory subunit B alpha isoform	2ABA_RAT	1.74
Kidney	Serine/threonine-protein phosphatase 2A 55 kDa regulatory subunit B alpha isoform	2ABA_RAT	1.74
Brain	Peroxiredoxin 2	PRDX2	−4.00
Brain	Enolase 1	ENO1	1.54
Brain	Chaperonin containing TCP1, subunit 2 (beta)	CCT2	2.04
Brain	Malate dehydrogenase 1	MDH1	1.43
Lung	Heat shock protein beta-1	HSPB1	5.77
Lung	Guanine deaminase	GDA	−2.40

**Table 5:** Significantly regulated proteins 7 days after hyperoxia (T7) in different organs. Positive values express up-regulation (*n* = 7), negative values down-regulation (*n* = 3).

Organ	Protein Name	Accession Number	IF at T7
Kidney	Microtubule-associated tumor suppressor 1 homolog	MTUS1_RAT	8.12
Kidney	40S ribosomal protein SA	RSSA_RAT	−1.77
Kidney	ATP synthase subunit alpha mitochondrial	ATPA_RAT	−1.81
	Voltage-dependent anionselective channel protein 1	VDAC1_RAT	
Kidney	Fructose-1,6-bisphosphatase 1	F16P1_RAT	1.74
	Actin, cytoplasmic 2	ACTG_RAT	
	Actin, cytoplasmic 1	ACTB_RAT	
Kidney	Serine/threonine-protein phosphatase 2A 55 kDa regulatory subunit B alpha isoform	2ABA_RAT	1.74
Kidney	Serine/threonine-protein phosphatase 2A 55 kDa regulatory subunit B alpha isoform	2ABA_RAT	1.74
Brain	Glucose-6-phosphate dehydrogenase	G6PD	23.91
Brain	Phosphatidylethanolamine- Binding protein 1	PEBP1	−4.54
Lung	Interferon-induced protein with tetratricopeptide repeats 3	IFIT3	2.72
Lung	Heat shock protein beta-1	HSPB1	6.79
